# Bypassing primary care facilities: health-seeking behavior of middle age and older adults in China

**DOI:** 10.1186/s12913-021-06908-0

**Published:** 2021-08-30

**Authors:** Changle Li, Zhuo Chen, M. Mahmud Khan

**Affiliations:** 1 Department of Health Economics, School of Health Management, Inner Mongolia Medical University, Hohhot, China; 2 Department of Health Policy and Management, College of Public Health, University of Georgia, 100 Foster Rd, Wright Hall 116, Athens, GA 30602 USA; 3 Centre for Health Economics, School of Economics, University of Nottingham Ningbo China, Ningbo, China

**Keywords:** Health-seeking behavior, Bypassing, Primary care, Older adults, China

## Abstract

**Background:**

With economic development, aging of the population, improved insurance coverage, and the absence of a formal referral system, bypassing primary healthcare facilities appear to have become more common. Chinese patients tend to visit the secondary or tertiary healthcare facilities directly leading to overcrowding at the higher-level facilities. This study attempts to analyze the factors associated with bypassing primary care facilities among patients of age 45 years or older in China.

**Methods:**

Random effects logistic models were used to examine bypassing of primary health facilities among rural-urban patients. Data from 2011 to 2015 waves of the China Health and Retirement Longitudinal Study were used.

**Results:**

Two in five older patients in China bypass primary health centers (PHC) to access care from higher-tier facilities. Urban patients were nearly twice as likely as rural patients to bypass PHC. Regardless of rural-urban residence, our analysis found that a longer travel time to primary facilities compared to higher-tier facilities increases the likelihood of bypassing. Patients with higher educational attainment were more likely to bypass PHCs. In rural areas, patients who reported their health as poor or those who experienced a recent hospitalization had a higher probability of bypassing PHC. In urban areas, older adults (age 65 years or older) were more likely to bypass PHC than the younger group. Patients with chronic conditions like diabetes also had a higher probability of bypassing.

**Conclusions:**

The findings indicate the importance of strengthening the PHCs in China to improve the efficiency and effectiveness of the health system. Significantly lower out-of-pocket costs at the PHC compared to costs at the higher tiers had little or no impact on increasing the likelihood of utilizing the PHCs. Improving service quality, providing comprehensive person-centered care, focusing on family health care needs, and providing critical preventive services will help increase utilization of PHCs as well as the effectiveness and efficiency of the health system.

**Supplementary Information:**

The online version contains supplementary material available at 10.1186/s12913-021-06908-0.

## Background

Health system of China established a three-tier healthcare delivery structure in both rural and urban areas since 1950s. The essential features of this system have remained quite stable even today with a network connecting healthcare providers at county, township, and village levels in rural areas, and at municipal, district, and community levels in urban areas [1]. Despite structural stability, the 1978 economic liberalization significantly affected the functioning of the health facilities at all levels. The multi-level referral system is now virtually absent [2, 3]. With the rapid demographic transition and aging of the population, the prevalence of chronic diseases and disabilities has increased [4]. Many local level primary centers (PHCs) are not ready to offer chronic disease management [5]. Another significant change was the adoption of a universal health insurance scheme that has provided coverage to 95% of the population since 2011 [6]. With rapid economic development, aging of the population, improved insurance coverage, and lack of formal referral structure, bypassing PHCs to seek care from secondary and tertiary healthcare facilities (STFs) have increased, leading to overcrowding at the higher-tier facilities [7].

Since the 2009 health reforms in China, significant investments were made to strengthen primary care infrastructure [7, 8]. Many PHCs were constructed and/or upgraded [9]. In 2019, there were approximately 9300 community health centers and 26,000 community health clinics in urban areas and 36,000 township health centers, and 622,000 village clinics in rural areas. Today, almost every community has at least one PHC [10]. In addition, to encourage utilization of PHCs, basic medical insurance schemes reimburse a higher proportion of costs to patients for seeking care from the PHCs rather than from STFs [11]. The zero-profit drug policy as part of the national essential drug system (NSDS) was introduced in PHCs in 2009. Under the NSDS, the PHCs must sell drugs at cost without any mark-up [12, 13]. Despite these financial incentives in favor of PHCs, there appears to be no decline in the propensity to bypass PHCs [8].

Bypassing the PHCs can adversely affect health system performance and contributes to higher medical expenditures [14]. People who bypass PHCs report spending approximately two times more on healthcare than those who do not bypass PHCs [15, 16]. Although bypassing of PHCs has become widespread, there is limited evidence on reasons and consequences of bypassing in China. Most studies on bypassing, conducted in selected countries in Asia, Africa, and the Americas, defined bypassing as incidents where people travel to another facility to obtain care rather than using the nearby local facility [17–23]. Few other studies have defined bypassing as skipping the PHCs to obtain care from STFs [24–26] without explicitly considering the distance to facilities from patients’ residences.

In the literature, several factors are found to be important in explaining the bypassing phenomenon. Important demographic and socioeconomic factors include age, gender, marital status, educational attainment, income, health insurance status, and health conditions. Sanders et al. [27], Shah [28], and Yao and Agadjanian [23] found that older patients are more likely to bypass local providers. However, Liu et al. [29], Escarce and Kapur [30], and Tai et al. [31] reported the opposite for patients in the USA. Liu et al. [29] found that patients who have some college education are less likely to bypass, but Akin and Hutchinson [17] and Damrongplasit and Wangdi [25] observed the opposite. Similarly, Liu et al. [29] and Perera and Weerasinghe [32] found a positive association between being married and bypassing, but Damrongplasit and Wangdi concluded to the contrary [25]. In the USA, like the Chinese health system, most insurance plans allow patients to seek care from upper-level providers or facilities without formal referrals.

Effects of gender, socioeconomic status, and health conditions on bypassing tendency were similar across several studies. Rao and Sheffel [26] and Akin and Hutchinson [17] found that females are more likely than males to bypass local providers. Rao and Sheffel [26] and Yao and Agadjanian [23] reported a higher likelihood of bypassing with increasing income or wealth. Radcliff et al. [33] found that uninsured patients have a lower tendency to bypass, and Akin and Hutchinson [17] and Tai et al. [31] found that bypassing local facilities increases with increasing severity of medical conditions. Sanders et al. [27], on the other hand, reported that bypassing is more common among patients with better overall self-rated health.

Provider characteristics associated with bypassing include size of the practice, availability of technology, service quality and mix, cost, and ownership. Escarce and Kapur [30] found that patients are less likely to bypass large local providers with extensive technological capabilities. Perceived poor quality of local healthcare providers increases bypassing (Paul and Rumsey [21], Kruk et al. [18], Liu, Bellamy et al. [20], and Sanders et al. [22]). Leonard et al. [34] reported that patients in Tanzania bypass health providers that overuse injections or overprescribe drugs. Patients with relatively low income may bypass local providers to seek care from less expensive providers [16]. Roh and Moon [35] and Escarce and Kapur [30] observed that patients in rural USA are more likely to bypass local public-sector providers to access care from private providers.

Patients are less likely to bypass local health care providers when the distance to alternative providers is relatively high (Tai et al. [31], Escarce and Kapur [30], and Leonard et al. [34]). Varkevisser and van der Geest [36] reported that distance is an important factor in the choice of hospitals, i.e., patients are less likely to bypass the nearest hospital when travel time to the next-nearest hospital increases.

Understanding the reasons for bypassing PHCs will help design government policies on how to make the PHCs more effective at the local level. Despite the importance of the topic, only a few studies have analyzed this phenomenon using large longitudinal datasets. This study is an attempt to understand the factors affecting the propensity to bypass PHCs in China despite the cost-advantages created by policy makers to encourage the utilization of PHCs.

## Methods

### Theoretical and empirical models

Patients have options in terms of health facilities they can use. For this analysis, the current study defines the options as either seeking care from the “primary care center” in the locality (PHC) or choosing an alternative “secondary or tertiary facility” (STF). Patients, in theory, will choose a facility-type that provides them a higher level of utility (*u*), i.e., if *u*_*i*_(*PHC*) < *u*_*i*_(*STF*), patient *i* is likely to seek care from secondary or tertiary health facilities. The literature suggests that the utility *u*(.) associated with the use of a health facility type may depend on several factors, including patient characteristics, illness type, facility characteristics, costs associated with seeking care, health system design. Since the choice has been defined as a dichotomous outcome, this study can develop the model by considering the likelihood of not selecting PHC [*p*(~*PHC*)]. Therefore, the generalized model of bypassing PHCs can be written as:

$$ p\left(\sim PHC\right)=f\left(Z,C,T,R|X\right) $$

where *X* is a vector of individual characteristics (age, gender, income); *Z* is facility-specific characteristics (e.g., facility size, technology availability, perceived quality); *C* is the out-of-pocket expenses associated with the facility-types, *T* is travel distances, and *R* represents other factors (e.g., physician recommendations on choice of facilities).

Given patient characteristics, choosing a health facility will require comparing the relevant factors between the local facility and other potentially accessible facilities. In China, utilization of PHCs will be less expensive than obtaining care from upper-level facilities and insurance coverage lowers the cost of using PHCs even further. Therefore, insurance status may be used as one of the proxy variables of financial incentives to access PHCs. Higher-tier facilities, by definition, provide more comprehensive healthcare services using better equipment and technology. Therefore, higher severity and relatively complex medical conditions may benefit from accessing care from higher-tier facilities. One important factor that affects the choice of facilities is their relative distances. If the closest STF is located relatively far away from a community, patients may elect to use PHC, keeping other factors constant. For empirical estimation, the ratio of the distances to PHC and STFs may be used to represent the relative degrees of accessibility of the facilities. This ratio is expected to be less than 1.0, but the higher the ratio, the greater should be the likelihood of bypassing.

For the analysis of bypassing behavior, the probability of not using PHC [*p*(~*PHC*)], the first step was to carryout simple descriptive analysis to understand the differences between patients who bypass PHCs (bypassers) and those who do not (non-bypassers). Pearson’s chi-square test was used to analyze the categorical independent variables. For the multivariate analysis, it is assumed that there is an unobserved latent variable ${y}_{it}^{\ast }$ affecting the decision to bypass.

$$ {y}_{it}^{\ast }={X}_{it}^{\prime}\alpha +{Z}_{it}^{\prime}\beta +{C}_{it}^{\prime}\gamma +{T}_{it}^{\prime}\theta +{\mu}_i+{\varepsilon}_{it},i=1,\dots, n,t=1,\dots, {T}_i $$

Where the vectors ${X}_{it}^{\prime },{Z}_{it}^{\prime },{C}_{it}^{\prime }, and\ {T}_{it}^{\prime }$ represent individual patient-specific, PHC specific, relative costs and relative distances associated with PHC and other secondary and tertiary health care facilities for individual *i* at time *t*, *α*, *β*, *γ and θ* are the estimated coefficients of covariates, *μ*_*i*_ is the unobserved and individual-specific heterogeneity, and *ε*_*it*_ is a time-varying error term. There is a binary variable *y*_*it*_ = [~*PHC*] where

$$ {y}_{it}=\left[\sim PHC\right]=1\  if\ {y}_{it}^{\ast }>0, and\ 0\  otherwise $$

where *y*_*it*_ = 1 indicates that the individual did not use PHC, i.e., bypassed the primary level of care.

The probability that *y*_*it*_ = 1, i.e., *P*(~*PHC*) can be written as

$$ {\displaystyle \begin{array}{c}\mathrm{P}\left(\sim \mathrm{PHC}\right)=P\left({y}_{it}=1\ \right|{X}_{it}^{\prime },{Z}_{it}^{\prime },{C}_{it}^{\prime },{T}_{it}^{\prime },\alpha, \beta, \gamma, \theta, {\mu}_i\left)=P\left({y}_{it}^{\ast }>0\ \right|{X}_{it}^{\prime },{Z}_{it}^{\prime },{C}_{it}^{\prime },{T}_{it}^{\prime },\alpha, \beta, \gamma, \theta, {\mu}_i\right)\\ {}=P\left({X}_{it}^{\prime}\alpha +{Z}_{it}^{\prime}\beta +{C}_{it}^{\prime}\gamma +{T}_{it}^{\prime}\theta +{\mu}_i+{\varepsilon}_{it}>0\ \right|\ {X}_{it}^{\prime },{Z}_{it}^{\prime },{C}_{it}^{\prime },{T}_{it}^{\prime },\alpha, \beta, \gamma, \theta, {\mu}_i\Big)\\ {}=P\left(-{\varepsilon}_{it}<{X}_{it}^{\prime}\alpha +{Z}_{it}^{\prime}\beta +{C}_{it}^{\prime}\gamma +{T}_{it}^{\prime}\theta +{\mu}_i\ \right|\ {X}_{it}^{\prime },{Z}_{it}^{\prime },{C}_{it}^{\prime },{T}_{it}^{\prime },\alpha, \beta, \gamma, \theta, {\mu}_i\Big)=F\left({\mu}_i+{X}_{it}^{\prime}\alpha +{Z}_{it}^{\prime}\beta +{C}_{it}^{\prime}\gamma +{T}_{it}^{\prime}\theta \right)\end{array}} $$

It assumes that the error term *ε*_*it*_ follows a logistic distribution as expressed below:

$$ {\displaystyle \begin{array}{c}P\left(\sim PHC\right)=P\left(\left({y}_{it}=1\ \right|\ {X}_{it}^{\prime },{Z}_{it}^{\prime },{C}_{it}^{\prime },{T}_{it}^{\prime },\alpha, \beta, \gamma, \theta, {\mu}_i\right)=\frac{e^{\mu_i+{X}_{it}^{\prime}\alpha +{Z}_{it}^{\prime}\beta +{C}_{it}^{\prime}\gamma +{T}_{it}^{\prime}\theta }}{1+{e}^{\mu_i+{X}_{it}^{\prime}\alpha +{Z}_{it}^{\prime}\beta +{C}_{it}^{\prime}\gamma +{T}_{it}^{\prime}\theta }}\\ {}P\left(\left({y}_{it}=0\ \right|\ {X}_{it}^{\prime },{Z}_{it}^{\prime },{C}_{it}^{\prime },{T}_{it}^{\prime },\alpha, \beta, \gamma, \theta, {\mu}_i\right)=\frac{1}{1+{e}^{\mu_i+{X}_{it}^{\prime}\alpha +{Z}_{it}^{\prime}\beta +{C}_{it}^{\prime}\gamma +{T}_{it}^{\prime}\theta }}\end{array}} $$

Furthermore, the present study assumes that *μ*_*i*_ is not related to the covariates. Correlation between *μ*_*i*_ and the covariates would indicate a fixed-effects model [37].

### Data source

The China Health and Retirement Longitudinal Study (CHARLS), conducted by the National School of Development of Peking University, is the data set used for this analysis. The sample of CHARLS was drawn from 450 villages or urban communities of 150 counties or equivalent urban districts in 28 provinces. A multistage stratified sampling with probability proportional to size was used for the survey. More details on the sampling and data collection process are available in Zhao et al. [38]. The CHARLS is nationally representative and collects data from persons of age 45 years or older and their spouses. The CHARLS respondents are reinterviewed every 2 years, with the first wave in 2011 covering 17,705 persons and two follow-ups in 2013 and 2015 with 18,605 persons and 21,095 persons, respectively. The CHARLS questionnaires include questions on household characteristics, demographic background, health status, and physical functioning, health care utilization, insurance status, work, retirement and pension, income, expenditure, asset ownership, housing characteristics, etc. From the datasets, only the individuals who sought outpatient care during the previous 4 weeks before the date of interview were selected. The final sample consisted of a total of 10,061 individuals in all three waves combined.

### Measures

#### Rural and urban definitions

In the CHARLS, each adult was asked, “What is the type of your address?” and “Is it a rural village or urban community?”. All respondents were classified as either rural or urban residents based on their responses.

#### Dependent variable

The PHCs in China provide generalist clinical care and basic public health services through community health centers and clinics in the urban areas and township hospitals and village clinics in the rural areas [13, 39]. In this study, bypassing PHCs has been defined as skipping any primary care facilities to obtain care from the secondary or tertiary level. Responses to the following survey questions were used to identify the incidence of “bypassing”: (1) Which healthcare provider did you visit most recently during the past 4 weeks? 1. General hospital; 2. Specialized hospital; 3. Chinese medicine hospital; 4. Community healthcare center; 5. Township hospital; 6. Community health clinic; 7. Village clinic/Private clinic; and 8. Other; and (2) Did the provider visit you at home? 1. Yes, and 2. No. If a respondent answered Question (1) by selecting any of General hospital, Specialized hospital, or Chinese medicine hospital and reported “No” on Question (2), the patient is considered to have had a “bypassing” of the PHC. The respondents who reported using “Other” providers in Question (1) or “Yes” to Question (2) were dropped from this study sample.

#### Independent variables

Independent variables were chosen based on theoretical considerations and literature review. The survey collected data on travel time to the facility for each of the patients. Therefore, those who obtained care from PHCs reported travel time to the PHCs, while those who visited the upper-level facilities (STFs) reported travel time to those facilities. Reported travel times to facility-types (PHCs or STFs) were used to predict the travel times to PHCs and STFs for all patients living in a community but did not visit the facility-type. The average travel time to PHCs for patients living in a community, for example, was used to approximate the travel time of those living in the same community but did not use the PHCs. The same method was used to predict the travel time from home to an STF for patients in each community. Relative travel time was defined as the ratio of travel time to a PHC to travel time to an STF.

China has two basic medical insurance schemes: Urban Employee Basic Medical Insurance and Urban and Rural Resident Basic Medical Insurance (the medical insurance of integrating Urban Resident Basic Medical Insurance and New Rural Cooperative Medical Scheme officially in 2016). Insurance status affects out-of-pocket expenses of patients differentially for seeking care from PHCs and STFs. Another critical factor affecting bypassing is patients’ medical conditions, especially the presence of chronic conditions. This analysis included an extensive list of chronic conditions, including hypertension, dyslipidemia, diabetes, cancer, chronic lung diseases, liver disease, heart diseases, stroke, emotional, nervous or psychiatric problems. Definitions of the variables are given in Table 1.

**Table 1:** Definitions of variables: Analysis of Bypassing Primary Health Centers, the China Health and Aging Longitudinal Study, 2011–2015

Variable	Description
**Dependent variable**	
Bypassing	1 if a patient bypasses primary health center (PHC) to seek care from a higher-tier facility (secondary or tertiary facilities); 0 otherwise.
**Independent variable**	
Age (years)	
45–54	1 if the individual is aged 45–54 years; 0 otherwise
55–64	1 if the individual is aged 55–64 years; 0 otherwise
≥ 65	1 if the individual is aged ≥65 years; 0 otherwise
Male	1 if the individual is male; 0 for female
Educational attainment	
Illiterate	1 if the individual is illiterate; 0 otherwise
Elementary school	1 if the individual attended elementary school; 0 otherwise
Middle school	1 if the individual graduated from middle school; 0 otherwise
High school	1 if the individual graduated from high school; 0 otherwise
Above three-years of college	1 if the individual had above three-years of college; 0 otherwise
Married	1 if the individual is married; 0 otherwise
Household income	
Low income	1 if the individual’s household income is in the first quartile; 0 otherwise
Lower middle income	1 if the individual’s household income is in second quartile; 0 otherwise
Upper middle income	1 if the individual’s household income is in the third quartile; 0 otherwise
High income	1 if the individual’s household income is in the highest quartile; 0 otherwise
Medical insurance	
UEMI	1 if the individual is enrolled in Urban Employee Medical Insurance; 0 otherwise
URMI	1 if the individual is enrolled in Urban Resident Medical Insurance; 0 otherwise
NRCMI	1 if the individual is enrolled in New Rural Cooperative Medical Insurance; 0 otherwise
Other Insurance	1 if the individual is enrolled in Government Medical Insurance, Urban and Rural, has either Resident Medical Insurance, Private Medical Insurance, Medical Aid, or Other medical insurance; 0 otherwise
No Insurance	1 if the individual does not have medical insurance; 0 otherwise
Health status	
Poor	1 if the individual reports health status to be poor; 0 otherwise
Fair	1 if the individual reports health status to be fair; 0 otherwise
Good	1 if the individual reports health status to be good or better; 0 otherwise
Specific chronic conditions	
Hypertension	1 if the individual has hypertension; 0 otherwise
Dyslipidemia	1 if the individual has dyslipidemia; 0 otherwise
Diabetes	1 if the individual has diabetes or high blood sugar; 0 otherwise
Chronic lung diseases	1 if the individual has chronic lung diseases; 0 otherwise
Heart diseases	1 if the individual has heart diseases; 0 otherwise
Kidney disease	1 if the individual has kidney disease; 0 otherwise
Stomach diseases	1 if the individual has stomach or other digestive diseases; 0 otherwise
Arthritis or rheumatism	1 if the individual has arthritis or rheumatism; 0 otherwise
Other major chronic diseases	1 if the individual has cancer or malignant tumor, liver disease, stroke, emotional, nervous or psychiatric problems, memory-related disease, or asthma; 0 otherwise
Functional limitations	
None	1 if the individual can eat, toilet, dress, bathe/shower, get in/out of bed, and walk without difficulty; 0 otherwise
Mild	1 if the individual has one or two difficulties in eating, toileting, dressing, bathing/showering, getting in/out of bed, or walking; 0 otherwise
Moderate	1 if the individual has three or four difficulties in eating, toileting, dressing, bathing/showering, getting in/out of bed, or walking; 0 otherwise
Severe	1 if the individual has five to six difficulties in eating, toileting, dressing, bathing/showering, getting in/out of bed, or walking; 0 otherwise
Hospitalization	1 if the individual reports hospitalization in the past 12 months; 0 otherwise
Relative travel time	The ratio of travel time to a primary care facility to travel time to a secondary or tertiary level facility for individuals in the community

### Data analysis

This analysis is based on a sample of 10,061 respondents who sought outpatient care during the 4 weeks prior to the survey date. Since follow-up visits may bias the results, this study has estimated the models separately for all visits (first visit and revisits) and the first visits. The sample size for first visits for an episode of illness was 4720. If the primary care providers in the first visits refer patients to specialists, the subsequent visits may reflect physician recommendations rather than patients’ decision-making. In other words, at least some of the revisits may not be considered bypassing of the PHCs.

Logistic regression models were employed to analyze factors affecting propensity to bypass PHCs stratified by rural-urban residence. Since access to health facilities differs significantly between rural and urban areas, the stratification is important to avoid potential bias created by rural-urban differences (see Table A1 of Additional file 1). The annex table also identifies a number of relevant variables for inclusion in the regression models. In most panel studies, the preferred empirical model would be either the fixed-effects or the random-effects logistic model [37] and empirical results should guide the choice between the model-types.

When estimating the patient fixed effects model, whenever patients bypass (or do not bypass) PHC in two waves or all three waves, by definition, variations over time becomes zero, and the empirical model will drop the cases. In the full sample of the fixed effects logistic models, 6620 out of 7672 rural patients and 2185 out of 2389 urban patients were dropped because of lack of inter-temporal variation. The random-effects model uses all the available observations and allows estimation of parameters and standard errors [40]. This study used Hausman’s specification test for the random effects logistic regression models to test the orthogonality of the random effects and the regressors [41]. The Hausman test suggests that the random-effects model is preferred over the fixed-effects model.

## Results

Among those who used outpatient services, the percent of patients bypassing PHCs were 34.0% in Wave 1, 39.4% in Wave 2, and 41.8% in Wave 3, indicating an increasing trend of bypassing over the years. In rural areas, the percent bypassing PHCs were 27.0% in Wave 1, 33.0% in Wave 2, and 34.6% in Wave 3. In contrast, the propensity to bypass PHCs remained almost static in urban areas over the years – 62.4% in Wave 1, 61.1% in Wave 2, and 60.1% in Wave 3. Urban patients were nearly twice as likely as rural patients to bypass PHCs. The results remained similar when only the first visits were considered, i.e., excluding the follow-up outpatient visits. Table A1 of Additional file 1 compares patient characteristics by bypassing behavior (patients bypassing PHCs vs. those not bypassing). The results indicate that bypassers differed from non-bypassers in terms of their residence, educational attainment, household income, medical insurance coverage, specific chronic conditions, and the incidence of hospitalization.

The odds ratios of the random effects logistic regression analysis are reported in Table 2. The results imply that rural patients aged 65 years or older were less likely to bypass PHCs than patients in the age group 45–54 years (OR = 0.64; *p* < 0.001). In contrast, patients aged 65 years or older show a higher probability of bypassing in urban areas (OR = 1.47; *p* = 0.036). The probability of bypassing PHCs increases with higher educational attainment in both rural and urban areas. Compared to illiterate patients, individuals who attended elementary school show increased odds of bypassing by 22% in rural areas (OR = 1.22; *p* < 0.012), and completion of more than 3 years of college increased the odds by 236% in urban areas (OR = 3.36; *p* < 0.001). Urban patients in the lower-middle income category were less likely to bypass PHCs than those with high income (OR = 0.56; *p* = 0.010). A similar trend was observed for patients in the upper-middle income category (OR = 0.69; *p* = 0.018). Rural patients enrolled in Urban Employee Basic Medical Insurance had three times the odds (OR = 3.01; *p* < 0.001) of bypassing PHCs than the uninsured. Compared with the uninsured patients, urban patients enrolled in New Rural Cooperative Medical Scheme had a lower odds of bypassing (OR = 0.47; *p* = 0.012).

**Table 2:** Random effects logistic regression results: bypassing primary health centers, the China Health and Aging Longitudinal Study, 2011–2015

	Rural			Urban		
	OR	95% CI	P	OR	95% CI	P
Age group						
45–54	1.00	Ref.		1.00	Ref.	
55–64	0.92	(0.78, 1.07)	0.273	1.12	(0.81, 1.54)	0.506
≥ 65	0.64	(0.53, 0.77)	< 0.001	1.47	(1.03, 2.11)	0.036
Male	1.11	(0.96, 1.27)	0.161	1.02	(0.78, 1.35)	0.871
Educational attainment						
Illiterate	1.00	Ref.		1.00	Ref.	
Elementary school	1.22	(1.04, 1.43)	0.012	1.30	(0.87, 1.94)	0.195
Middle school	1.57	(1.27, 1.93)	< 0.001	1.97	(1.27, 3.05)	0.002
High school	1.87	(1.38, 2.54)	< 0.001	2.82	(1.73, 4.59)	< 0.001
> three-years of college	1.76	(0.51, 6.10)	0.373	3.36	(1.77, 6.37)	< 0.001
Married	1.09	(0.89, 1.33)	0.397	1.05	(0.72, 1.54)	0.798
Household income						
Low income	0.84	(0.69, 1.02)	0.081	0.84	(0.58, 1.20)	0.332
Lower middle income	0.87	(0.72, 1.05)	0.156	0.56	(0.36, 0.87)	0.010
Upper middle income	0.95	(0.79, 1.15)	0.602	0.69	(0.50, 0.94)	0.018
High income	1.00	Ref.		1.00	Ref.	
Medical insurance						
UEMI	3.01	(1.73, 5.22)	< 0.001	1.52	(0.87, 2.67)	0.140
URMI	1.49	(0.72, 3.06)	0.283	1.62	(0.87, 3.00)	0.125
NRCMI	0.92	(0.68, 1.24)	0.576	0.47	(0.26, 0.85)	0.012
Other Insurance	1.10	(0.60, 2.00)	0.775	1.03	(0.59, 1.78)	0.929
No Insurance	1.00	Ref.		1.00	Ref.	
Health status						
Poor	1.34	(1.16, 1.54)	< 0.001	1.28	(0.94, 1.73)	0.113
Fair				1.00	Ref.	
Good	1.22	(0.99, 1.51)	0.063	0.98	(0.68, 1.41)	0.900
Specific chronic conditions						
Hypertension	0.95	(0.81, 1.11)	0.492	0.82	(0.61, 1.11)	0.195
Dyslipidemia	1.24	(0.99, 1.56)	0.061	1.01	(0.71, 1.43)	0.959
Diabetes	1.13	(0.86, 1.48)	0.374	1.76	(1.15, 2.70)	0.009
Chronic lung diseases	0.87	(0.71, 1.05)	0.155	0.98	(0.65, 1.47)	0.906
Heart diseases	1.40	(1.15, 1.70)	0.001	1.24	(0.87, 1.76)	0.228
Kidney disease	0.81	(0.64, 1.02)	0.073	0.70	(0.44, 1.12)	0.138
Stomach diseases	0.88	(0.77, 1.02)	0.087	0.93	(0.68, 1.28)	0.662
Arthritis or rheumatism	0.83	(0.72, 0.95)	0.006	0.89	(0.66, 1.20)	0.442
Other major chronic diseases	0.88	(0.73, 1.06)	0.192	0.64	(0.44, 0.91)	0.015
Functional limitations						
None	1.00	Ref.		1.00	Ref.	
Mild	0.93	(0.79, 1.10)	0.401	1.06	(0.73, 1.54)	0.745
Moderate	1.02	(0.76, 1.35)	0.907	1.19	(0.57, 2.46)	0.643
Severe	1.44	(0.95, 2.17)	0.085	1.54	(0.55, 4.32)	0.408
Hospitalization	2.08	(1.77, 2.45)	< 0.001	2.15	(1.53, 3.02)	< 0.001
Relative travel time	1.15	(1.13, 1.17)	< 0.001	1.28	(1.18, 1.39)	< 0.001
Constant	0.25	(0.17, 0.38)	< 0.001	0.63	(0.30, 1.30)	0.207
Observations	7672			2389		

Rural patients with poor health were more likely to bypass PHCs than those who reported their health being ‘fair’ (OR = 1.34; *p* < 0.001). Patients with heart diseases in rural areas and urban patients with diabetes had a higher odds of bypassing PHCs (OR = 1.40; *p =* 0.001, OR = 1.76; *p* = 0.009), and the opposite was true for rural patients with arthritis or rheumatism and patients with other major chronic conditions in urban areas (OR = 0.83; *p* = 0.006, OR = 0.64; *p* = 0.015). Patients who experienced hospitalization in the past 12 months had a higher odds of bypassing in both the rural and urban areas (OR = 2.08; *p* < 0.001, OR = 2.15; *p* < 0.001). Relative travel time was positively associated with patients bypassing PHCs in rural and urban areas (OR = 1.15; *p* < 0.001, OR = 1.28; *p* < 0.001).

Table 3 presents factors affecting bypassing PHCs when only the first contact outpatient visits are used in the estimation. The results are similar to the estimates obtained when all visits, including the revisits, were used in the analysis.

**Table 3:** Random-effects logistic regression results: bypassing primary health centers for new visits (excluding revisits for the same episode) by older adults, the China Health and Aging Longitudinal Study, 2011–2015

	Rural			Urban		
	OR	95% CI	P	OR	95% CI	P
Age group						
45–54	1.00	Ref.		1.00	Ref.	
55–64	1.08	(0.85, 1.39)	0.488	1.02	(0.69, 1.50)	0.932
≥ 65	0.91	(0.68, 1.21)	0.509	1.71	(1.07, 2.73)	0.024
Male	1.03	(0.84, 1.29)	0.727	1.00	(0.71, 1.40)	0.998
Educational attainment						
Illiterate	1.00	Ref.		1.00	Ref.	
Elementary school	1.21	(0.95, 1.55)	0.120	1.69	(1.00, 2.85)	0.050
Middle school	1.62	(1.17, 2.24)	0.004	2.16	(1.21, 3.85)	0.009
High school	1.89	(1.16, 3.06)	0.010	2.52	(1.34, 4.76)	0.004
> three-years of college	5.56	(0.97, 31.81)	0.054	2.94	(1.26, 6.86)	0.013
Married	1.07	(0.78, 1.47)	0.681	1.02	(0.63, 1.65)	0.936
Household income						
Low income	1.04	(0.77, 1.40)	0.808	1.01	(0.64, 1.60)	0.959
Lower middle income	1.02	(0.76, 1.36)	0.921	0.63	(0.36, 1.11)	0.110
Upper middle income	1.11	(0.83, 1.48)	0.498	0.73	(0.49, 1.09)	0.129
High income	1.00	Ref.		1.00	Ref.	
Medical insurance						
UEMI	1.53	(0.62, 3.75)	0.354	1.55	(0.76, 3.16)	0.227
URMI	2.84	(0.90, 8.96)	0.075	1.40	(0.65, 3.02)	0.387
NRCMI	0.86	(0.54, 1.36)	0.517	0.58	(0.28, 1.19)	0.136
Other Insurance	1.10	(0.33, 2.17)	0.726	0.86	(0.44, 1.68)	0.664
No Insurance	0.84	Ref.		1.00	Ref.	
Health status						
Poor	1.59	(1.25, 2.02)	< 0.001	1.03	(0.69, 1.56)	0.872
Fair				1.00	Ref.	
Good	1.34	(0.99, 1.81)	0.056	0.99	(0.65, 1.51)	0.972
Specific chronic conditions						
Hypertension	1.02	(0.79, 1.31)	0.897	0.93	(0.63, 1.37)	0.705
Dyslipidemia	1.63	(1.12, 2.37)	0.010	0.94	(0.60, 1.47)	0.778
Diabetes	0.93	(0.57, 1.50)	0.758	1.96	(1.00, 3.83)	0.049
Chronic lung diseases	0.96	(0.70, 1.31)	0.790	1.04	(0.61, 1.80)	0.876
Heart diseases	1.20	(0.86, 1.68)	0.291	1.40	(0.87, 2.25)	0.172
Kidney disease	0.59	(0.39, 0.89)	0.013	0.67	(0.36, 1.25)	0.210
Stomach diseases	0.88	(0.70, 1.11)	0.281	1.02	(0.70, 1.50)	0.911
Arthritis or rheumatism	0.79	(0.64, 0.98)	0.035	0.96	(0.67, 1.37)	0.807
Other major chronic diseases	0.88	(0.64, 1.20)	0.413	0.63	(0.37, 1.05)	0.075
Functional limitations						
None	1.00	Ref.		1.00	Ref.	
Mild	1.06	(0.80, 1.41)	0.662	1.07	(0.65, 1.77)	0.783
Moderate	1.47	(0.92, 2.34)	0.107	1.69	(0.49, 5.87)	0.410
Severe	1.91	(0.82, 4.46)	0.133	1.52	(0.31, 7.44)	0.603
Hospitalization	1.96	(1.47, 2.60)	< 0.001	1.47	(0.91, 2.36)	0.113
Relative travel time	1.31	(1.24, 1.39)	< 0.001	1.31	(1.16, 1.48)	< 0.001
Constant	0.15	(0.07, 0.30)	< 0.001	0.47	(0.19, 1.17)	0.104
Observations	3653			1067		

## Discussion

The nationally representative survey indicates that the proportion of outpatients bypassing PHCs increased over the years in China. Urban patients were nearly twice as likely to bypass PHCs than rural patients. This is not unexpected due to the easy access and availability of many STFs in urban areas. Travel distance and travel-related costs are likely to be lower for the urban population [36, 42]. Rural patients often must rely on PHCs in the area due to the difficulty of accessing alternative health care providers. Especially for rural residents, obtaining care from upper-level facilities may become quite expensive due to higher out-of-pocket expenses and longer travel times [43].

About two in five older adults in China bypassed PHCs to obtain care from upper-level providers (STFs). This is despite significantly lower out-of-pocket costs for services and drugs at PHCs than at the STFs. Therefore, financial incentives have not been adequate to discourage bypassing of PHCs in China. The results imply that propensity to bypass PHCs is unlikely to decline significantly if the financial incentives in favor of PHCs are made even stronger. Given that the bypassing tendency is related to age, rural/urban location, present of chronic conditions, etc., it points to supply-side concerns, probably related to availability and quality of service-mix offered at the PHCs.

The random-effects logistic regression model identified several factors affecting bypassing of PHCs for rural and urban residents separately. The estimates suggest that factors affecting the first visits are very similar to factors affecting all outpatient visits (both new and follow-up visits), implying that physician recommendations were not important in influencing the choice of facility-types. Regardless of rural-urban residence, a relative increase in travel time (ratio of travel times to PHC and STF) increased the probability of bypassing PHCs. The result is consistent with the findings in the literature, i.e., increased travel time lowers the utilization of health care facilities [33, 44–50]. Without a formal referral system, in urban areas, where health resources are much richer and STFs are more concentrated than those in rural areas in China, urban patients are more likely to bypass PHCs because of easy accessibility and availability of medical care services they need.

Patients with higher educational attainment show a higher probability of bypassing PHCs. Educational attainment is an indicator of socioeconomic status (SES) as well as knowledge about the relative quality of services facilities offer. Middle-aged and older adults with higher level of income are also more likely to bypass PHCs. This probably indicates perception of lower quality of services at the PHCs compared to the STFs. With an increase in income and educational attainment, patients become more sensitive to service quality and less sensitive to cost savings that can be achieved by utilizing PHCs.

In rural areas, self-reported poor health status increased the likelihood of bypassing PHCs. Again, this possibly reflects a lack of confidence in the quality of services and/or non-availability of individualized services at the PHC [51, 52]. Offering individualized services is a person-centered care approach [53]. Patients who experienced hospitalization in the past 12 months are more likely to bypass PHCs as the hospitalization in the recent past may be associated with actual or perceived higher severity of illness or need for individualized services, encouraging utilization of STFs [29]. A number of chronic conditions also affect the choice of facility-type. Patients with arthritis or rheumatism were less likely to bypass PHCs probably because these conditions make travel more difficult [54], especially for rural patients who must travel significantly longer distances to reach STFs.

Older adults show high degree of bypassing PHCs in urban areas but for rural areas, the opposite was true (middle-aged individuals more likely to bypass than the older adults). This tendency may be associated with lack of relevant services at the PHC level. Older adults require more personalized care and continuity of care is important for their health status. In contrast, for rural areas, social value of protecting the health of middle-aged individuals may be higher and travel to a STF is more costly and difficult for the elderly. Among urban patients, older adults were more likely to bypass PHCs than those aged 45–54 years. Geriatric services have not been properly incorporated at the primary level in urban China [55], which may explain urban older adults’ hesitancy to seek care from the PHCs. Urban patients with diabetes are also more likely to use upper-level facilities, again reflecting the lack of chronic disease management at the primary level [56].

In urban areas, where the upper-level facilities are easily accessible, the tendency to bypass is significantly higher. With improvements in health status and the aging of the population, patients need individualized as well as continuity of care services. Unless those services are offered from the PHCs, bypassing problem is expected to increase further [57, 58].

There are several limitations of this study. The CHARLS survey provides information only on the most recent outpatient service utilized in the previous 4 weeks. Measuring the incidence of bypassing using the most recent outpatient visits within the last 4 weeks may bias the results. The survey did not collect information on facility and provider characteristics, and the study could not analyze the effect of provider characteristics on the choice of health facilities.

## Conclusions

About two in five older adults in China bypassed PHCs to obtain care from upper-level providers (STFs). Urban patients were twice as likely as rural patients to bypass PHCs. The present study found that relative increase in travel time and more educated had a higher likelihood of bypassing primary care in rural and urban areas. Moreover, poor health status and being hospitalized in the past 12 months had a higher likelihood of bypassing primary care in rural areas. On the other hand, arthritis or rheumatism decreased the odds of bypassing. Furthermore, older age and diabetes had a higher likelihood of bypassing primary care in urban areas.

Continuity of care and individualized attention require redesigning the mode of care delivery at the PHC level with emphasis on patient-provider trust and adoption of proactive health promotion and disease prevention. Structural changes, including the staffing of the PHCs, will be needed to make the PHCs more effective in addressing the needs of the elderly and middle-aged individuals. The findings of the study indicate the importance of strengthening the service provision in rural PHCs to address common chronic conditions. In fact, improving the service delivery for addressing the needs of chronic conditions will improve utilization of PHCs in both rural and urban areas.

In conclusion, improving the utilization of PHCs in China will require improving the quality of services rendered from these facilities. Perceived quality of medical care delivered from the PHCs can be improved if the facilities are able to offer comprehensive person-centered health care, focusing on the family health care needs, and making critical screening and preventive services available. Availability of services alone will not improve overall quality of services – it will require a system of continuity of care by ensuring good rapport between patients and physicians. If patients feel that they have their own primary care physician in the PHCs, the likelihood of seeking care from PHCs should be significantly higher.

## Supplementary Information

**Additional file 1.**

## Data Availability

The dataset used for drafting the paper is a publicly available dataset available in the Peking University Open Research Data Platform repository. The dataset is downloadable for research purposes through the link: https://opendata.pku.edu.cn/dataverse/CHARLS.
