# Supplementary material for: Bypassing primary care facilities: health-seeking behavior of middle age and older adults in China
Source: BMC Health Serv Res. 2021 Aug 30;21:895. doi: 10.1186/s12913-021-06908-0 (PMC8406824; doi:10.1186/s12913-021-06908-0)
Supplement: Supplementary file 1 — Additional file 1. [file 12913_2021_6908_MOESM1_ESM.docx]

**Additional file 1**

**Table A1 Characteristics of patients bypassing and non-passing primary health centers**

|  | **Wave 1 (2011)**  N=2,981 | | **Wave2 (2013)**  N=3,643 | | **Wave 3 (2015)**  N=3,437 | |
| --- | --- | --- | --- | --- | --- | --- |
|  | **Non-bypassers**  N=1,967 | **Bypassers**  N=1,014 | **Non-bypassers**  N=2,208 | **Bypassers**  N=1,435 | **Non-bypassers**  N=2,000 | **Bypassers**  N=1,437 |
| **Overall** (%) | 65.98 | 34.02 | 60.61 | 39.39 | 58.19 | 41.81 |
|  |  |  |  |  |  |  |
| Residence (%) |  |  |  |  |  |  |
| Urban | 11.29 | 36.29 | 14.58 | 35.26 | 19.35 | 40.64 |
| Rural | 88.71 | 63.71 | 85.42 | 64.74 | 80.65 | 59.36 |
| **P value** | <0.001 | | <0.001 | | <0.001 | |
| Age group (%) |  |  |  |  |  |  |
| 45-54 | 29.54 | 35.70 | 29.44 | 32.26 | 33.80 | 32.57 |
| 55-64 | 38.38 | 36.19 | 35.24 | 36.10 | 32.45 | 34.10 |
| ≥65 | 32.08 | 28.11 | 35.33 | 31.64 | 33.75 | 33.33 |
| **P value** | 0.002 | | 0.051 | | 0.574 | |
| Gender (%) |  | |  | |  | |
| Male | 40.77 | 46.25 | 42.03 | 42.16 | 40.30 | 46.07 |
| Female | 59.23 | 53.75 | 57.97 | 57.84 | 59.70 | 53.93 |
| **P value** | 0.004 | | 0.937 | | 0.001 | |
| Educational attainment |  |  |  |  |  |  |
| Illiterate | 35.03 | 21.10 | 32.79 | 21.32 | 29.30 | 20.53 |
| Elementary school | 42.35 | 38.66 | 42.53 | 37.00 | 45.01 | 38.48 |
| Middle school | 15.46 | 22.19 | 17.57 | 22.72 | 17.81 | 23.11 |
| High school | 6.30 | 13.61 | 5.98 | 15.19 | 7.02 | 13.48 |
| > 3-years of college | 0.86 | 4.44 | 1.13 | 3.76 | 0.86 | 4.39 |
| **P value** | <0.001 | | <0.001 | | <0.001 | |
| Married (%) |  |  |  |  |  |  |
| Yes | 84.04 | 89.25 | 84.28 | 85.30 | 85.55 | 87.40 |
| No | 15.96 | 10.75 | 15.72 | 14.70 | 14.45 | 12.60 |
| **P value** | <0.001 | | 0.407 | | 0.119 | |
| Household income (%) |  |  |  |  |  |  |
| Low income | 28.27 | 19.43 | 28.35 | 20.77 | 24.35 | 26.44 |
| Lower middle income | 27.25 | 20.32 | 27.76 | 20.63 | 28.35 | 22.06 |
| Upper middle income | 23.79 | 27.42 | 23.23 | 27.94 | 26.80 | 23.87 |
| High income | 20.69 | 32.84 | 20.65 | 30.66 | 20.50 | 27.63 |
| **P value** | <0.001 | | <0.001 | | <0.001 | |
| Medical insurance (%) |  |  |  |  |  |  |
| UEMI | 5.34 | 18.84 | 6.57 | 22.16 | 3.60 | 14.20 |
| URMI | 2.24 | 8.09 | 3.49 | 7.11 | 0.90 | 2.64 |
| NRCMI | 84.39 | 63.12 | 83.56 | 61.67 | 79.40 | 58.25 |
| Other Insurance | 2.59 | 6.80 | 2.85 | 4.81 | 10.25 | 17.88 |
| No Insurance | 5.44 | 3.16 | 3.53 | 4.25 | 5.85 | 7.03 |
| **P value** | <0.001 | | <0.001 | | <0.001 | |
| Health status (%) |  |  |  |  |  |  |
| Poor | 47.94 | 49.21 | 42.98 | 43.76 | 40.45 | 42.52 |
| Fair | 41.03 | 38.56 | 45.43 | 43.55 | 48.15 | 45.44 |
| Good | 11.03 | 12.23 | 11.59 | 12.68 | 11.40 | 12.04 |
| **P value** | 0.355 | | 0.436 | | 0.292 | |
| Hypertension |  | |  | |  | |
| Yes | 29.38 | 33.43 | 27.63 | 26.97 | 24.05 | 28.25 |
| No | 70.62 | 66.57 | 72.37 | 73.03 | 75.95 | 71.75 |
| **P value** | 0.023 | | 0.663 | | 0.005 | |
| Dyslipidemia |  | |  | |  | |
| Yes | 10.83 | 18.05 | 10.01 | 14.77 | 10.40 | 14.47 |
| No | 89.17 | 81.95 | 89.99 | 85.23 | 89.60 | 85.53 |
| **P value** | <0.001 | | <0.001 | | <0.001 | |
| Diabetes |  | |  | |  | |
| Yes | 7.12 | 13.51 | 5.93 | 9.76 | 6.85 | 8.70 |
| No | 92.88 | 86.49 | 94.07 | 90.24 | 93.15 | 91.30 |
| **P value** | <0.001 | | <0.001 | | 0.044 | |
| Chronic lung diseases |  | |  | |  | |
| Yes | 16.68 | 14.69 | 13.63 | 12.75 | 14.80 | 12.94 |
| No | 83.32 | 85.31 | 86.37 | 87.25 | 85.20 | 87.06 |
| **P value** | 0.162 | | 0.445 | | 0.122 | |
| Heart diseases |  | |  | |  | |
| Yes | 15.86 | 23.18 | 13.04 | 17.14 | 13.60 | 17.75 |
| No | 84.14 | 76.82 | 86.96 | 82.86 | 86.40 | 82.25 |
| **P value** | <0.001 | | 0.001 | | 0.001 | |
| Kidney disease |  | |  | |  | |
| Yes | 9.91 | 11.34 | 8.65 | 7.94 | 9.65 | 91.65 |
| No | 90.09 | 88.66 | 91.35 | 92.06 | 90.35 | 8.35 |
| **P value** | 0.226 | | 0.452 | | 0.192 | |
| Stomach diseases |  | |  | |  | |
| Yes | 35.13 | 30.57 | 30.21 | 28.22 | 33.30 | 28.74 |
| No | 64.87 | 69.43 | 69.79 | 71.78 | 66.70 | 71.26 |
| **P value** | 0.013 | | 0.199 | | 0.004 | |
| Arthritis or rheumatism |  | |  | |  | |
| Yes | 46.92 | 41.12 | 40.58 | 35.61 | 43.85 | 36.53 |
| No | 53.08 | 58.88 | 59.42 | 64.39 | 56.15 | 63.47 |
| **P value** | 0.003 | | 0.003 | | <0.000 | |
| Other major chronic diseases |  | |  | |  | |
| Yes | 18.10 | 19.53 | 15.35 | 14.98 | 16.05 | 16.35 |
| No | 81.90 | 80.47 | 84.65 | 85.02 | 83.95 | 83.65 |
| **P value** | 0.342 | | 0.761 | | 0.812 | |
| Functional limitations (%) |  |  |  |  |  |  |
| None | 75.18 | 77.71 | 74.18 | 75.75 | 73.55 | 75.02 |
| Mild | 17.45 | 14.20 | 19.11 | 17.28 | 19.60 | 17.81 |
| Moderate | 5.29 | 4.64 | 4.98 | 4.74 | 4.70 | 5.22 |
| Severe | 2.09 | 3.45 | 1.72 | 2.23 | 2.15 | 1.95 |
| **P value** | 0.016 | | 0.370 | | 0.517 | |
| Hospitalization (%) |  |  |  |  |  |  |
| Yes | 12.76 | 23.57 | 19.88 | 29.06 | 18.15 | 28.11 |
| No | 87.24 | 76.43 | 80.12 | 70.94 | 81.85 | 71.89 |
| **P value** | <0.001 | | <0.001 | | <0.001 | |
| Relative travel time |  |  |  |  |  |  |
| Ratio<=1 | 83.02 | 74.26 | 75.72 | 76.59 | 82.45 | 44.12 |
| Ratio>1 | 16.98 | 25.74 | 24.28 | 23.41 | 17.55 | 55.88 |
| **P value** | <0.001 | | 0.522 | | <0.001 | |
